# Security Issues and Software Updates Management in the Industrial Internet of Things (IIoT) Era

**DOI:** 10.3390/s20247160

**Published:** 2020-12-14

**Authors:** Imanol Mugarza, Jose Luis Flores, Jose Luis Montero

**Affiliations:** Industrial Cybersecurity, IKERLAN Technology Research Center, Basque Research and Technology Alliance (BRTA), P.J.M. Arizmendiarrieta 2, 20500 Arrasate/Mondragón, Spain; jlflores@ikerlan.es (J.L.F.); jlmontero@ikerlan.es (J.L.M.)

**Keywords:** security, maintenance, issues, incidents, software updates, patches

## Abstract

New generation Industrial Automation and Control Systems (IACS) are providing advanced connectivity features, enabling new automation applications, services and business models in the Industrial Internet of Things (IIoT) era. Nevertheless, due to the extended attack surface and increasing number of cyber-attacks against industrial equipment, security concerns arise. Hence, these systems should provide enough protection and resiliency against cyber-attacks throughout their entire lifespan, which, in the case of industrial systems, may last several decades. A sound and complete management of security issues and software updates is fundamental to achieve such goal, since leading-edge security countermeasures implemented in the development phase may eventually become out-of-date. In this article, a review of the IEC 62443 industrial security standard concerning the security maintenance of IIoT systems and components is given, along with guidelines for the implementation of such processes. As concluded, the security issues and software updates management shall jointly be addressed by the asset owner, service providers and product suppliers. These security processes should also be compatible with the safety procedures established by safety standards.

## 1. Introduction

The Industrial Internet of Things (IIoT), an industrial subset of the Internet of Things (IoT) model, are enabling new industrial control and automation applications, services and business models. In this technological trend, referred to as the fourth industrial revolution or Industry 4.0, high inter-connectivity among all sensors, devices, machines and people, is envisioned, with the aim of improving automation, productivity and self-monitoring. The need of human intervention for process diagnostics, analysis and decision-making is also reduced.

In this context, security emerges as an essential requirement to protect this working environment, in which according to Kaspersky [1], more than 150 industrial control systems-related vulnerabilities are discovered every year. Appropriate security measures and procedures are typically implemented during the development period of the industrial control system. However, as stated by Bruce Scheiner, an internationally renowned security technologist, “Security is a process, not a product” [2]. Due to the long operational periods, the security countermeasures implemented and adopted at the development phase of the system might become obsolete at some point. Throughout this long-term period, different security-related flaws and weaknesses might be discovered (i.e., an outdated cryptographic scheme). These security issues may be internally originated (for example, the detection of a design flaw by a developer and/or tester) or externally discovered (for example, by third-party security researchers or customers).

It may not be easy to understand the differences between safety and security terms. Actually, just a single word is given for both concepts in some languages, for example, as in Spanish, Swedish or German. In this way, the linguistics do not help to clarify and distinguish these two concepts [3]. As described by the International Atomic Energy Agency (IAEA) [4], security tries to reduce malicious risks, prevent attacks and misuses in order to protect assets. In contrast, safety attempts to prevent accidents and incidents, which could impact on peoples health or damage to the environment. While safety incidents and accidents are involuntary, security ones are usually originated by on-purpose malevolent attacks. These events are unexpected and not easy to predict beforehand [5]. The combination of safety and non-safety-critical applications, such as security, is defined as a mixed-criticality system. In such systems, a strong isolation among applications is crucial [6]. In safety, the goal is fault containment, namely, the propagation of the fault through the system. In security, the consequences of misuses or malicious intrusions and attacks are kept under control.

**Figure 1:**
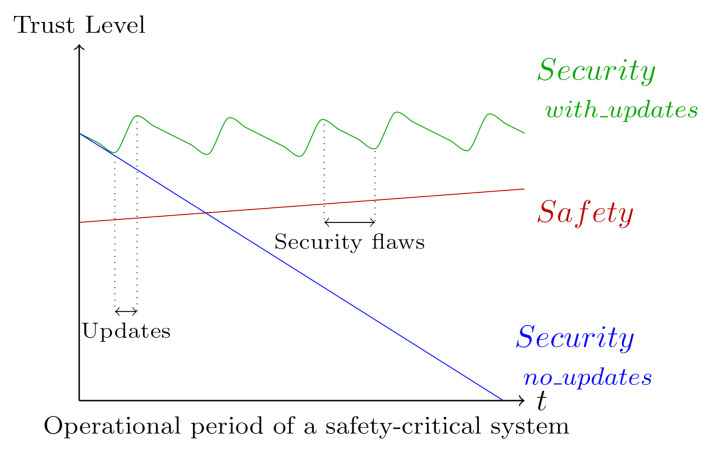
Safety and security trust levels [7,8,9,10].

As shown in Figure 1, in contrast to the security domain, which may require performing updates periodically in order to maintain the security level, the trust level on traditional technologies involved in industrial control systems increases with the passage of time, especially in safety-related systems.

Well-known and solid technologies and methods are commonly employed in safety-engineering, which are further tested, verified and validated through time. A safety and security co-engineering is then essential [11,12]. To this end, the International Electromechanical Commision (IEC) has developed two technical reports: IEC TR 63069 [13] and IEC TR 63074 [14]. As stated by the IEC TR 63069 technical document, “the communication and interaction between the safety and security domains should be implemented throughout the life cycle” [13]. Security experts should review any modification made in a safety-related system, since it may affect the results of the security risk assessment. On the contrary, security-related software updates should not be installed until a safety impact analysis is carried out. Safety and security processes should, respectively, be managed according to the IEC 61508 [15] and IEC 62443 [16] standards [13].

In this article, an analysis of the industrial security IEC 62433 standard [16] for the security issues and software updates management on IIoT systems and components is provided. In addition, guidelines for the specification, implementation and enforcement of security-related procedures are also provided. This article is structured as follows: after this introduction, an overview of the IEC 62443 industrial cybersecurity standard is provided. Following, procedures and guidelines for the security issues and software update management, based on the requirements of the IEC 62443 standard [16], are provided. Recommendations given by the IEC TR 63069 [13] and IEC TR 63074 [14] are also considered. Finally, conclusions are presented and future lines and open issues discussed.

## 2. Overview of IEC 62443

The ISA/IEC-62443 is a series of standards, technical reports, and related information that define procedures and requirements for implementing electronically secure Industrial Automation and Control Systems (IACS) [16]. This standard, created by the International Society of Automation ISA, was originally named ISA-99. Nevertheless, it was renumbered to ISA-62443 in 2010. The purpose of this modification was to align ISA documents with the analogous IEC standards.

As expressed by this standard, security risk management shall jointly and collaboratively be addressed by all entities involved in the design, development, integration, and maintenance of the industrial and/or automation solution (including subsystems and components) to achieve the required security level. In this sense, the standard provides the description of the involved stakeholders in the design, development and maintenance of secure IIoT systems and components, which are:

- Product supplier: “Manufacturer of secure hardware and/or software product”, such as an embedded controller or a networking device. These products, which shall be developed following a secure development lifecycle, might be a single component or a group of components acting together as a system or subsystem.
- Service provider: “Individual or organisation (internal or external organisation, manufacturer, etc.) that provides a specific support service and associated supplies in accordance with an agreement with the asset owner”. It integrates the product suppliers products into an industrial automation solution for the asset owner. It may also provide system maintenance services.
- Asset owner: The individual or organisation (internal or external organisation, manufacturer, etc.) that owns and is responsible for a physical and/or logical object having either a perceived or actual value to it.

**Figure 2:**
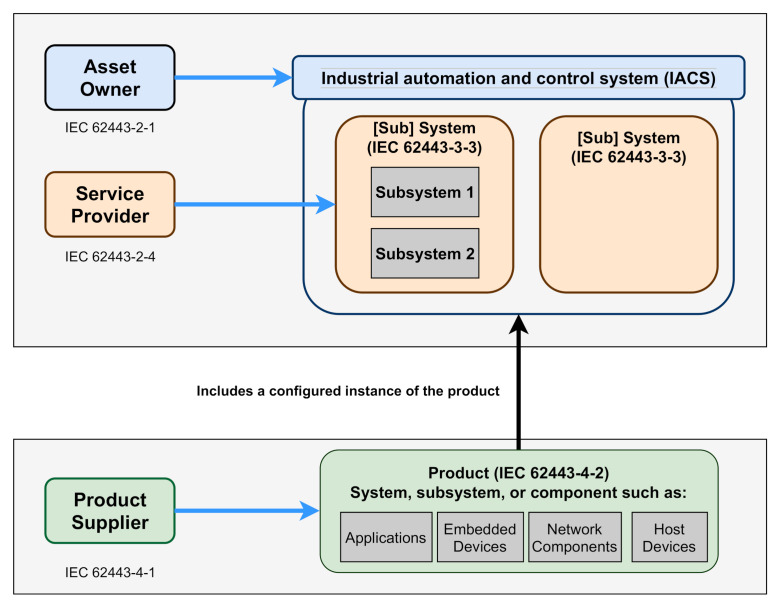
Overview of IEC 62443 [16].

Figure 2 illustrates the joint strategy for industrial automation and control systems (IACS).

This joint effort is reflected in the organisation of the documents of the standard. Four general categories are defined, which are *General*, *Policies and Procedures*, *System* and *Component*:

1. General: Provides background information such as security concepts, terminology and metrics;
2. Policies and procedures: Addresses the security and patch management policies and procedures;
3. System: Provides system development requirements and guidance;
4. Component: Provides product development and technical requirements, intended for product vendors.

The overall security management requirements specified in these documents are based on the requirements included in the ISO 27000 series [17]. In fact, a mapping of requirements between IEC 62443-2-1 to ISO 27000 series [17,18] is provided in such document. Unlike ISO 27000, the IEC 62443 [16] standard, similarly to the functional safety IEC 61508 [15] standard, defines four different security levels (SL). Depending on the selected target security level, in the same way as in the safety domain, more demanding cyber-security requirements shall be fulfilled by IIoT systems and components. Table 1 provides an overview and characteristics of each of the established levels.

In addition, this standard recommends the adoption and implementation of the well-known defence-in-depth strategy [19]. This security concept consists of a design strategy and pattern in which multiple layers of security are defined and implemented throughout a system. These protection barriers provide multiple, usually concatenated, security protections, with the aim of preventing and/or delaying any cyber-attack. Cyber-attackers would then have to break through and/or bypass several security countermeasures. Assets are allocated in different security zones, depending on the required risk level and protection. Zones are “grouping of logical or physical assets that share common security requirements”. On the contrary, security conduits are “logical grouping of communication assets that protects the security of the channels it contains”.

All involved organisations and entities (asset owner, service providers and product suppliers) are highly encouraged to adopt and implement the ISO 9001 [20,21] quality management and ISO 27000 series [17,18] information security management standards, which can facilitate the adoption of this standard and its integration with other organisational management systems. It is worth mentioning that due to defined entities, which involve different individuals and organisations, security-related measures related to supply chain and third-party suppliers should be defined and enforced. To this end, the ISO 27036 [22], as recommended by IEC 62443, may be applied.

## 3. National Standards

Although the IEC 62443 standard [16] is considered the reference industrial security standard, other national standards (usually based on the ISO 27000 framework [17]) may also be considered and enforced depending on the current security legislation in force in each country. In this section, a comparison of several national standards and IEC 62443 will be provided. The most representative country in terms of cyber-security is the United States of America.

The North American Electric Reliability Corporation (NERC), which is the regulatory body for the energy industry in the United States, sets the cybersecurity standards for protecting America’s electrical infrastructure against cyber threats. The NERC Critical Infrastructure Protection (CIP) program [23] aims to identify and protect national critical infrastructures and assets associated with energy supply. The IEC 62443 security management program is highly compatible with this CIP program. The CIP standard documents associated to the security management are shown in Table 2.

The *U.S. Homeland Security department* presented the Cyber Resilience Review (CRR) methodology [24] for the analysis, assessment, and management of cybersecurity in critical infrastructures. This methodology is derived from the CERT Resilience Management Model (CERT-RMM) proposed by the Software Engineering Institute (SEI) at Carnegie Mellon University. The IEC 62443 covers, broadly speaking, around 75% of the aspects considered in the CERT-RMM methodology.

It is also worth mentioning that the SP800-82 special publication published by the NIST document [25,26] provides recommendations and best practices for the security of industrial control systems, including, for example, SCADA (Supervisory Control And Data Acquisition) systems, DCS (Distributed Control System) and other control system configurations, such as PLCs (Programmable Logic Controllers). This technical document also identifies common security threats and vulnerabilities in the scope of industrial systems and provides recommended security countermeasures to mitigate the associated risks.

As far as Europe is concerned, the Bundesamt für Sicherheit in der Informationstechnik (BSI), is the German federal agency responsible for the cybersecurity of computer and communication equipment. This agency published four standard documents related to the cyber-security maintenance: *BSI Standard 100-1 Information Security Management Systems (ISMS)* [27] defines the general requirements for the Information Security Management Systems (ISMS), fully compatible with ISO 27001. *BSI-Standard 100-2: IT-Grundschutz Methodology* [28] describes the steps to be taken to implement and operate the previously presented ISMS in practice. *BSI-Standard 100-3: Risk Analysis based on IT-Grundschutz* [29] provides a cyber security risk analysis methodology, which could be used within the IT-Grundsschtz framework, that is for the ISMS. Finally, *BSI-Standard 100-4: Business Continuity Management* [30] describes a systematic approach to the development, adoption and maintenance of a business continuity management system at the organisational level. In addition, the OLF 104:2016 [31], published by the Norwegian Oil Industry Association, defines the cyber-security requirements for control processes, safety, and industrial control systems. This standard references the ISO/IEC 27002 and ISO/IEC 27031 documents [17,18] and IEC 62443 can cover half of the relevant aspects. The Spanish *Esquema Nacional de Seguridad* cyber-security program [32,33] (originally designed for public administration) also takes into consideration such standard.

Finally, it should be pointed out that the cyber-security guides published by Dutch *Netherlands Organisation for Applied Scientific Research* and French *Agence nationale de la sécurité des systèmes d’information* agencies make direct reference to international standards, for example, ISO 27000. Generally speaking, most national cyber-security standards focus on the requirements and implementation of the information security management system.

Table 3 provides a mapping of IEC 62443-2-3 contents to national USA and Europe standards. As observed in Table 3, the establishment of the security program proposed by IEC 62443 is aligned with national security frameworks, hence, reducing compliance efforts and duplicate processes. Nevertheless, the requirements for installation and maintenance suppliers are barely covered by national standards. As stated in [34], standards usually consider industrial automation and control systems installed in isolation within an individual organisation, but software and components are supplied by several vendors. Services from external service suppliers are also hired.

## 4. IIoT System Security Lifecycle

An IIoT system consists of a set of distributed and interacting services deployed across diverse devices to fulfil a series of industrial functions. An IIoT system will be formed upon different hardware or software components. In the industry 4.0 landscape, in which high inter-connectivity is envisioned, cyber-security has emerged as a constant issue to deal with, which needs to be specifically addressed and managed. To this end, different industrial cyber-security management methodologies and frameworks have been proposed [38]. A survey of cyber-security management in industrial systems was presented by W. Knowles et al. [38]. Nevertheless, at such time (2015), IEC 62443 was still under development.

According to this standard (IEC 62443-1-1 document [16]) a system is defined as the “interacting, interrelated, or interdependent elements forming a complex whole”, in which a component will be “one of the parts that make up a product or system. A component may be hardware or software and may be subdivided into other components” [39]. This taxonomy facilitates cyber-security management and responsibilities allocation for all involved stakeholders (asset owners, service providers and product suppliers).

**Figure 3:**
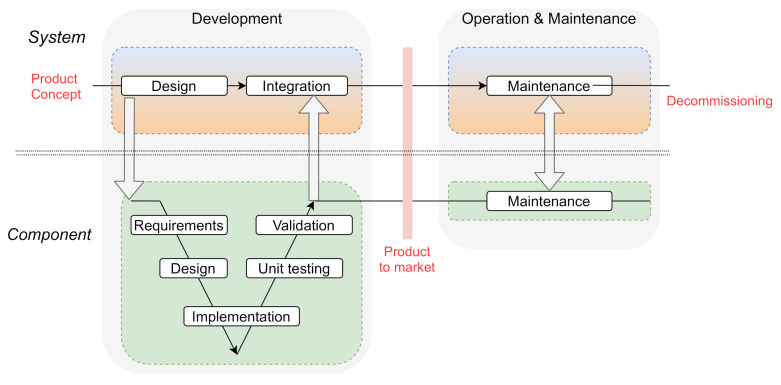
IIoT system and components of a simplified lifecycle.

Figure 3 depicts the system and components lifecycle and associated entities. As can be observed, the lifecycle is divided into two clear stages: the *development* stage (represented as the V model) and the *operational and maintenance* stage. Throughout these stages, the corresponding security manuals and guidelines should also be created. It has to be noted that although a V model development methodology is depicted in Figure 3, other methodologies could be used, such as *spiral* or *agil* [40]. The asset owner and service providers could integrate already existing standard commercial products in their system design, or call for customised ones developed specifically for their system.

During the system and components maintenance stage (in which security issues and software updates management processes should be executed), the asset owner, service providers and product suppliers might receive any kind of notifications about security-related events affecting and/or compromising the operated IIoT systems and components. These security events and notifications, which will trigger the IIoT system and components security-related maintenance activities, may include:

- Modifications to the IIoT system or components,
- Changes to the physical operation or environmental security measures.
- Discovery of a new vulnerability or bug embedded in the IIoT system or components.
- Release of a new application or operating system software patch.
- Scheduled periodic security reviews and audits.

With the aim of ensuring long-term business goals, an efficient and sound security program shall be defined and enforced by the asset owner and service providers and product suppliers. Following, on the one hand, requirements and guides for the definition of a system security maintenance program to be implemented by the asset owner is provided, according to IEC 62443 [16] international security standard. On the other hand, security-related integration and maintenance requirements established for service providers and product suppliers are given.

### 4.1. System Security Maintenance Program

The asset owner should design and establish a cyber-security management system (CSMS). For this purpose, the IEC 62443-2-1 [35], which defines the elements necessary to establish CSMS for industrial automation and control systems, might be applied. As stated by this technical document, the asset owner shall establish a cyber-security management program. This program, which is strongly based on the well-known ISO 27001 [17,18] and tailored to the industrial needs, establishes security policy, procedure, practice and personnel related assignments. The cyber-security management program elements are assorted in three main categories:

- Risk analysis: This category provides background organisation information as well as the identification of security risks that the organisation faces.
- Risk addressing: This category provides the methods, procedures and policies for the mitigation of previously identified risks.
- Continuous monitoring and improvement: This category provides continuous security management program processes.

**Figure 4:**
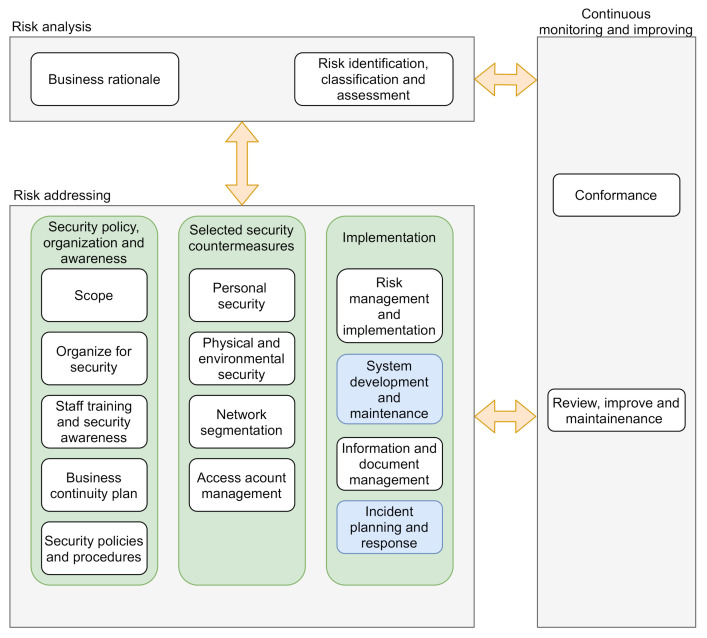
Elements of the cyber-security management program.

The elements of the cyber-security program defined by the IEC 62443-2-1 technical document [35] associated to the IIoT systems and components security maintenance are: *System development and maintenance* and *Incident planning and response*. Figure 4 provides a graphical overview of such program. The elements of the cyber-security program defined by the IEC 62443-2-1 technical document [35]. Elements associated to the IIoT systems and components security maintenance are highlighted in blue colour in Figure 4. The definition and enforcement of such security management program may imply a considerable effort that must be carefully studied. Likewise, an incremental establishment of such program is commonly recommended.

On the one hand, the *system development and maintenance* element ensures that the desired security level is maintained through the entire operational life of the IIoT system, from development, commissioning, until decommissioning. This cyber-security management element also takes into consideration the maintenance of cyber-security procedures and policies. On the other hand, the *incident planning and response* cybersecurity management element defines how all the cybersecurity-related incidents will be handled. This incident management element should also specify how the incident investigation and review will be reviewed, the IIoT system recovery strategies and documentation guidelines.

Furthermore, in order to sustain the security level of a given zone, the asset owner should regularly conduct a security review and a risk assessment activity *risk identification, classification and assessment* element, in which the impact of the observed security-related events in the IIoT system should be evaluated. It shall be verified that the initially determined security level of the IIoT system is still achieved and maintained through the adoption and/or implementation of different security measures. In case a new security protection mechanism is selected, a redefinition of the IIoT system architecture and network design might be required. It has to be clarified that the *review, improve and maintenance* element depicted in Figure 4 refers to the continuous review, improvement and maintenance of the cyber-security management program itself. These activities are highly related with ISO 9001 [20,21] quality management processes.

**Figure 5:**
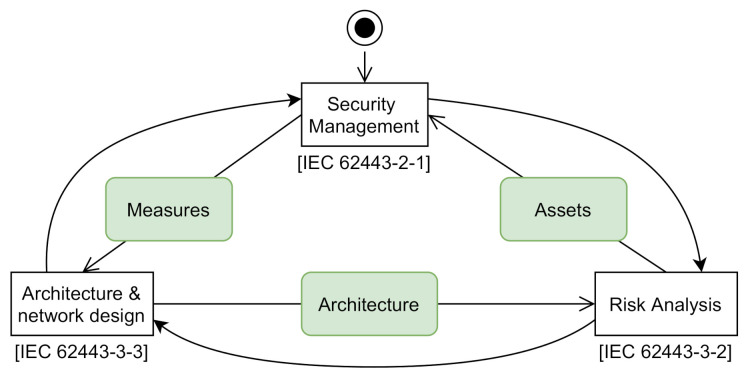
Security management, system architecture and network design, and risk analysis relationship.

Figure 5 shows the relationship between the security management processes and procedures (described in the cybersecurity program), the system architecture and network design, and the system risk analysis activity. As a result of the risk analysis activity, a catalogue of assets to be protected will be obtained, which is a list of all resources (facilities, people, documents, hardware, software, etc.), of value to the organisation. This information will be used as input for the *risk identification, classification and assessment* element (see Figure 4). After that, organisational and technical measures will be implemented, adopted and enforced to make sure that the identified security risks are addressed. These countermeasures may also imply a system architecture and network design modification, such as the integration of a firewall.

An asset inventory and management system is then essential to keep track of such assets and provide the actual value estimation throughout the operation and maintenance stage of the IIoT system. This catalogue should include all the systems and components within the scope of the organisation, including embedded controllers and all other industrial devices. Although focused on financial services, the NIST SP 1800-5 [41] provides a guide for assets tracking, configuration management and cyber-security. Manual assets management is commonly a costly activity and also prone to human errors. Therefore, an automated asset management is usually recommended. In this line, as established by the IEC 62443-4-1 document [39] (requirement SM-3), product suppliers should, for example, by means of a catalogue, identify their own products to which cyber-security is being addressed. The provided security level should also be indicated. This information should be supplied then provided to the service providers and asset owners.

### 4.2. Maintenance Services and Product Suppliers

As far as security maintenance services are concerned, the IEC 62443-2-4 technical document [37] specifies the requirements for the integration and maintenance activities for service providers. They shall ensure that all personnel and activities associated and involved in the system maintenance processes comply with the security policies, procedures and responsibilities established by the organisation. A security training and background check for such personnel is also needed. These measures will reduce the associated security threats and risks, such as the use of illegitimate USB memory sticks. Two different profiles are defined by IEC 62443 [37]:

- Integration service providers: provides capabilities to design and deploy an industrial control and automation solution for the asset owner.
- Maintenance service providers: executes system maintenance activities according to the asset owner’s needs.

The security maintenance is part of the overall system maintenance, which may also include regular equipment review, predictive maintenance, etc. It has to be noted that the maintenance service providers, which might be part of the asset owner’s organisation, for example, an independent department, are responsible for performing the following security maintenance activities throughout the lifecycle (not limited to):

- Patching and anti-virus updates;
- Equipment upgrades and maintenance;
- Component and (sub)system migrations;
- Change management;
- Remote access management;
- Contingency plan management (including backups and restores);
- Issues management.

Regarding product suppliers, the IEC 62443-4-1 technical document specifies the secure product development lifecycle requirements, both for the development and the maintenance phases (see Figure 3). The presented lifecycle is based (among others) on the Common Criteria [42,43] and the functional safety IEC 61508 [15] standard. The defined security-aware lifecycle is divided among eight practices.

The product supplier shall keep track of all products/components that have been developed and shall be maintained from the security point of view (requirement SM-3). Also, similarly to service providers, the product supplier shall guarantee that all personnel involved in the secure product development and/or maintenance has received security training and proved enough security expertise to perform the assigned tasks (requirement SM-4). The IEC 62443-4-1 document specifies that all security-related issues (reported by internal or external sources) shall be received, reviewed, addressed and tracked to closure. As guidance, the standard references the ISO 30111 [44] and ISO 29147 [45] standards. Requirements related to the handling of security-related issues are described in *Practice 6*.

On the contrary, *Practice 7* describes the processes and procedures to be applied for the products security updates management. It shall be ensured that the software update does not introduce regressions, and that it is released in a timely manner to product users, i.e., service providers. Finally, as stated by IEC 62443-4-1 [39], “a process shall be employed for verifying that a product or a patch is not released until its security-related issues have been addressed and tracked to closure” (requirement SM-11 in Practice 1).

## 5. Security Issues Management

Throughout the operational period of the industrial system, different security-related issues might show up, such as new discovered security-related bugs, vulnerabilities, design flaws or the release of new software patches (usually fixing a given software weakness). A process for receiving, evaluating and addressing these security-related issues is then needed. Each of the entities involved in the security maintenance of the system (asset owner, service providers and product suppliers) shall define and implement the required security issues management processes and procedures in their cyber-security maintenance programs. The correct dimensioning of the security issues management team and internal resources dedicated to it may vary considerably from sector to sector. For example, in the railway domain, Kour et al. [46] provide a statistical review of cyber-security incidents in the transportation sector. As shown, the number of cyber-security incidents related to transportation infrastructure has increased in the last years, in which the most common issue is the spreading of malware.

**Figure 6:**
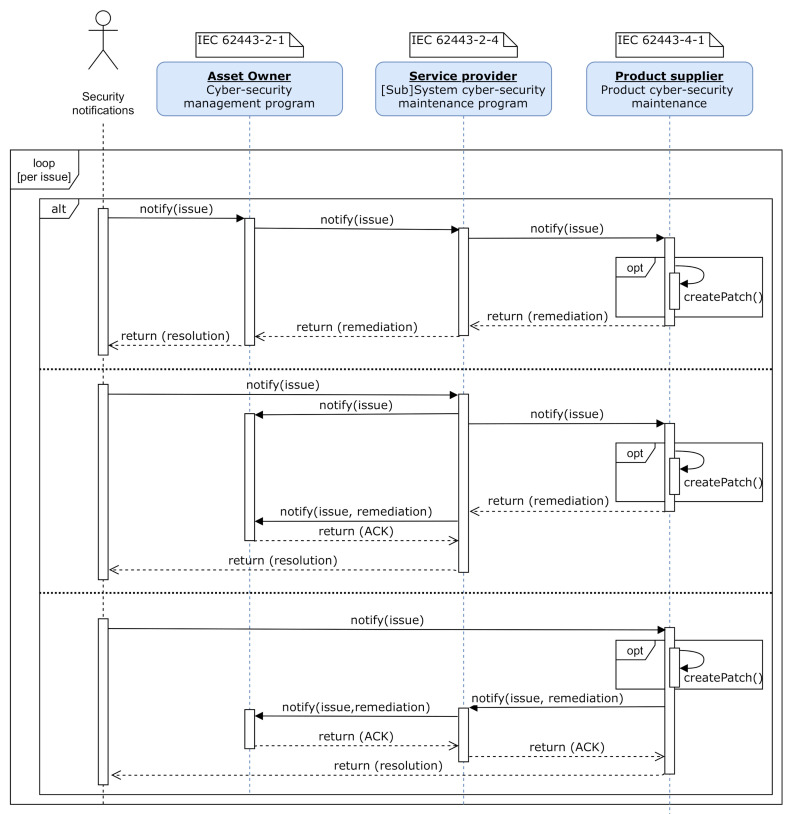
Security-related notifications and treatment sequence diagram.

Figure 6 illustrates the interaction between all involved organisations and entities defined by the IEC 62443 [16] standard. As depicted, the security maintenance management of the IIoT system and components shall jointly be addressed by the asset owner, service providers and product suppliers.

As recommended by IEC 62443-4-1 [39], the ISO 30111 [44] international standard might be adopted for security-related events, incidents and vulnerability management. In fact, the phases defined by ISO 30111 [44] are simply aligned with the IEC 62443-4-1 security issues management requirements. Table 4 shows this alignment. The definition and design of this process shall periodically be reviewed in order to come up with a more efficient, sound and complete process (requirement *DM-6*).

The ISO 30111 [44] standard, in contrast to IEC 62443, defines an initial *Preparation* phase, which refers to the definition of the management process itself. At this point, the security incident management procedures are defined and established. The ISO 27035 standard [47] also provides guidelines for information security events, incidents and vulnerabilities management [48,49]. It has to be noted that these steps should be accomplished in a timely manner, usually following the markets needs.

Following, each of the phases of *Practice 6* [39] for security issues handling are specified. For a successful and efficient completion of such tasks, the use of a security issues management tool is highly advised for tracking from receiving to closing the identified issues. In this line, an analysis of existing guidance frameworks focusing on cyber-incidents response and recovery is provided by A. Staves et al. [50]. However, due to paywall restrictions, only parts IEC 62443-2-1 [35] and IEC 62443-4-2 [51] of the documents were analysed. The IEC 62443-4-1 [39] document, in which the requirements and process description for security issues management are specified, was not evaluated.

### 5.1. Receiving

New security-related issues, as shown in Figure 6, will be received from different third-party entities, both internals or externals (i.e., product developers and testers, security researchers or product users). To this end, organisations should provide means for receiving such information in a secure manner (i.e., product support department or website). Typical reporting mechanisms are [45]: web forms, bug/issue tracking systems, vulnerability reporting services or e-mails. According to the North American Reliability Corporation (NERC) [23,52], in such new issue report, the following information should be provided (if possible) in order to, later on, facilitate verification and validation:

- Attack Vector: A description of the path or means by which the attacker compromise or attempt to compromise;
- Impact: A description of the functional impact for the compromise or attempt to compromise;
- Intrusion level: A description of the level of intrusion for the compromise or attempt to compromise.

The organisation will, through time, identify common security-related incidents and attack attempt patterns. As examined by Al-Mhiqani, Mohammed Nasser, et al. [53], in which different security-related incidents in industrial control systems were analysed, most of the cyber-attacks targeted system disruptions and sensitive data access, being cyber-war and cyber-crime the main purposes. As claimed by NIST [25], the industrial control system security objectives usually follow as the next priorities: availability, integrity and confidentiality.

### 5.2. Reviewing

In this phase, the collected security issues are reviewed, to verify and determine that the information and claims included in the received documentation are reproducible and accurate. This process may also involve the reproduction, in a security testing laboratory, of the reported vulnerability, exploit and/or incident. The security threats associated to the issue should be analysed.

In case the security weaknesses or the vulnerability can not be reproduced and verified, the organisation should request more information to the informer. Once verified, the applicability of the issue should be evaluated. It may be the case that the security issue is associated with an obsolete product (not supported any more by the organisation) or it is indeed already being addressed by the internal incident response team. Finally, the organisation should also notify and report the informer about already carried out analysis.

### 5.3. Assessing

Once the security-related incident is reviewed, its potential impact in the corresponding product should be analysed (note that, as shown in Table 4 this task is also included in the *verification* phase defined by ISO 30111 [44]). The potential impact will be determined upon diverse attributes, such as severity of the issue, affected products, number of affected devices deployed in the field, collateral damage or availability of exploits [39]. The categorisation of security-related issue shall be performed. For this purpose, an update of the initially accomplished security risk analysis and assessment might be performed (requirement SR-2 [39] in secure product development lifecycle). Alternatively, several security incidents scoring systems exist [54], for example, the Cyber Incident Scoring System (NCISS) proposed by NERC (https://us-cert.cisa.gov/nciss/demo), which is currently used by some USA federal agencies for the security issues classification. Additionally, a root cause analysis should be carried to determine (if exists) any underlying causes of security weaknesses and vulnerabilities, for example, by applying the IEC 62740 standard [55].

### 5.4. Addressing

Organisations should develop and test remediations for the already received, reviewed and assessed security incidents. According to IEC 63069 [13] and IEC 62443-4-1 [39], the incidence response might include the following actions as possible remediation measures:

- Software patching [13];
- Controlled power off [13];
- Deactivation of certain functions or parts of the system [13,39];
- System concept, architecture or defence in depth strategy change [13,39];
- Implementation of organisational procedures and/or measures [13];
- Use of compensating mechanisms [39], such as new security functions/capabilities.

As observed, the system software might not always be upgraded, specially while dealing with outdated or legacy systems. Moreover, configuration changes could be performed to address the security weakness and/or vulnerability. In some cases, the organisation may also decide not to fix the problem if the assessed residual risk is below the established acceptable risk level. Along with the technical remediation, organisations should upgrade the corresponding documentation and development recommendations.

### 5.5. Disclosing

Lastly, in the last step phase, the managed security-related issue is made public to product users and/or any other related stakeholders, such as to a Computer Emergency Response Team (CERT). In the disclosing report, the main key points and analysis information obtained through the execution of previous security issues handling phases shall be included. The implemented remediation or mitigation measures, such as the release of a software patch, should also be described.

Moreover, the organisation may inform privately to the corresponding third-parties with the goal of establishing a time period in which a remediation to the discovered security issue is designed prior to the public disclosure of it. This period is denoted *Embargo period* by ISO 29147 [45]. To this end, a secure communication security issue notification channel is necessary. For vulnerability disclosing, vulnerability advisories are published, sometimes in batches and schedule releases [45]. It is worth mentioning that a single vulnerability advisory might address multiple security weaknesses and vulnerabilities. The definition of the organisation’s disclosure policy might be challenging. Kulikova et al. [56] propose a decision-support framework for the definition of disclosure strategies.

## 6. Software Updates Management

Software updates are commonly applied to fix a given bug or improve the usability of the computer program. As of today, software patches related to industrial control systems have generally addressed stability and functionality issues rather than security [11]. Nevertheless, as stated by the IEC 62443 industrial cybersecurity standard [16], a patch management is an element of a complete and sound cyber-security strategy. It should be pointed out that “applying patches is a risk management decision” [16]. The software upgrade may be rejected or delayed if the cost to apply the patch is greater than the risk evaluated cost. The requirements and guidelines given by the IEC 62443 [16] standard are aligned with the industrial control systems patching recommendations provided by the USA Department of Homeland Security [57]. In addition, in the automotive domain, legal and regulatory requirements concerning software updates are being established at the time of writing [58,59].

**Figure 7:**
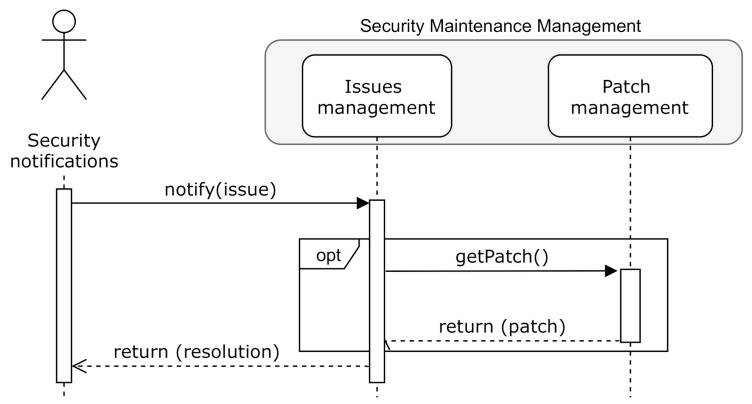
Security-related issues and patches management relationship.

Figure 7 depicts the relation between the security issue and patches management processes. As previously indicated, software updates will be evaluated in the *Remediation development* (following ISO 30111 [44]) or *Addressing* (following IEC 62443-4-1 *Practice 6* [16]) phase.

The *IEC 62443-2-3: Patch management in the IACS environment* technical document provides patch management related guidelines. Two different guidances on patching are provided. On the one hand, the asset owner guidance on patching describes four major patch management activities, which are: *Information gathering*, *Project planning and implementation*, *Procedures and policies for patch management* and *Operating a patch management system*. These tasks should be included in the *System development and maintenance* CSMS element. On the other hand, a reference procedure to develop and distribute new software updates for product suppliers is provided. This guidance defines four major activities: *Discovery of vulnerabilities*, *Development of security updates*, *Distribution of security information* and *Communication and outreach*.

**Figure 8:**
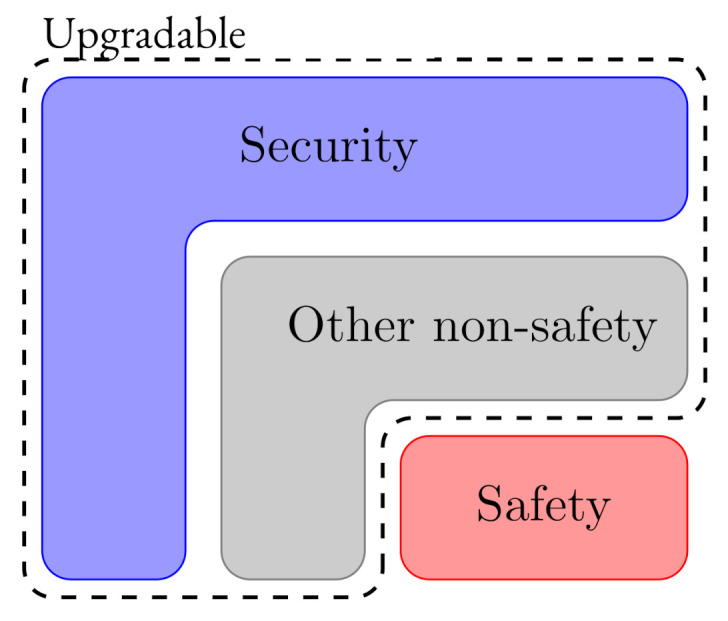
Upgradable type of software components.

In accordance with the IEC TR 63069 [13] and IEC TR 63074 [14] technical documents, the security-related software update management should be compatible with safety processes [15]. As manifested by IEC 62443 “applying patches is a risk management decicion”. Consequently, organisations should properly define which software modules of the IIoT components will be maintained and upgraded when necessary. Figure 8 illustrates the groups of updatable software modules installed in the system.

As stated by IEC TR 63069 [13], security software updates should not be applied to the safety-system without prior safety impact analysis. Usually, if physical environmental conditions do not change, safety-related updates are not necessary. Additional security measures, such as new firewall rules, could be adopted for legacy systems.

Following, the patch lifecycle model proposed by the IEC 62443 standard [16] is described. This model, which will be followed by the asset owner and service providers, starts with the delivery of a given software patch. After that, software patch generation and delivery considerations are presented, specially focused on update delivery timings.

### 6.1. Patch Lifecycle Model

**Figure 9:**
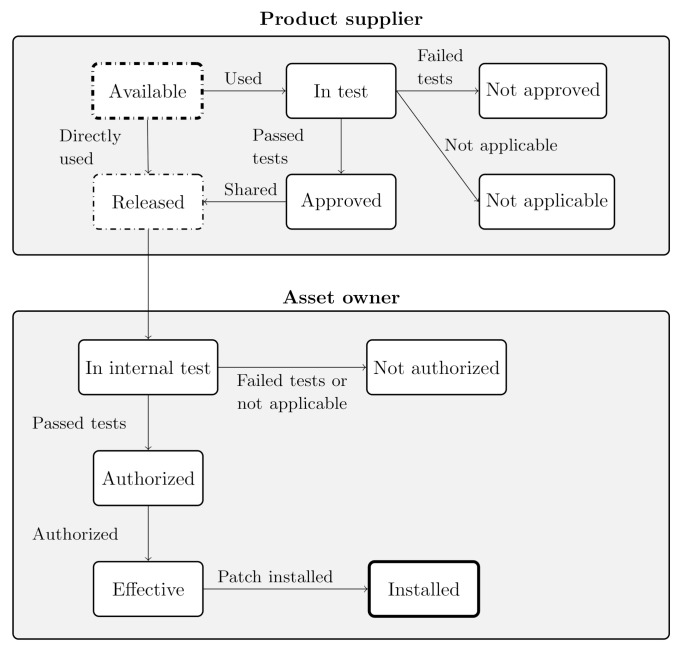
Patch lifecycle model [11,16].

The IEC 62443-2-3 [16] standard defines a patch lifecycle, which specifies a series of states through which a patch passes from the time that it is available by a third party or a product supplier until it is installed or rejected by the asset owner or the service provider. On the one hand, Figure 9 depicts the patch lifecycle state model, which is divided into two main parts. The first part corresponds to the states maintained by the product supplier. The product supplier might integrate in their IIoT components other types of SW modules (e.g., operating systems, communications stacks). On the contrary, the second part conforms to the states associated with the asset owner. The service providers and asset owners might be able to directly acquire all available and released patches, share or distribute them. That’s why the *Available* and *Released* states are depicted as dashed elements. As observed, the service provider entity is excluded from the patch lifecycle model. Maintenance service providers (if any) will accomplish system maintenance activities on behalf of the asset owner.

Table 5 gives the description of each of the states defined in the patch lifecycle model depicted in Figure 9. As shown in Figure 9 and described in Table 5, not all available updates will be approved and installed in the target. Clear and sound evidence will be required to ensure that the IIoT system will behave correctly functionally once the patch is applied. This activity is performed in the *In internal test* phase. In case all tests have successfully passed, the installation and/or application of the software patch is authorised. The software patch installation (corresponding with the **Installed** patch state depicted in Figure 9) is the process of modifying and/or updating the actual software of the system, usually to add minor software enhancements, provide compatibility features or fix security bugs.

The asset owner should try to minimise the resources spent dealing with patch testing and installation processes. For this purpose, an optimised patch release and installation strategies are required [60,61,62,63]. In these cost and risk models, operational downtime periods should be considered. However, as described by the IEC 62443-4-2 document [51], in case an industrial component is executing essential functions, patching and updating capabilities that do not compromise the availability properties of such functions are needed (requirements EDR 3.10, HDR 3.10, NDR 3.10). For this purpose, a software framework enabling live updates in industrial control systems is necessary [7,8,9,10,64]. These frameworks employ redundancy and multi-version execution techniques to avoid systematic faults.

### 6.2. Patch Generation and Delivery

As observed in Figure 6, the product supplier is responsible for the cyber-security maintenance of IIoT components, and therefore, creating the corresponding software patches. Specifically, the IEC 62443-4-1 document [39], which defines the requirements for the software patch generation and release, shall be followed. This process corresponds with the patch states depicted in Figure 9 corresponding with the product supplier.

Once a vulnerability or a security flaw is discovered, for example, a buffer overflow, the developer should fix the corresponding software part/function, and after that, create the new software version and the patch. This software patch should be then systematically evaluated to firstly ensure, that indeed, it addresses the discovered security flaw and secondly, to guarantee that it does not introduce regressions and it does not compromise any other system property, such as safety or availability (the update may cause serious operational disruptions). The compatibility of the new software with dependent components and/or operating system should also be evaluated. Similarly, if a new operating system security update is released, the consistency between the application and the upgraded operating system should be evaluated. All this software patch evaluation process shall be properly documented.

As far as the software update delivery is concerned, the product supplier should define a software update delivery policy, which determines the time-frames for evaluation and delivering security-related software updates. This policy may vary depending on diverse technical and business factors. As stated by M. Souppaya and K. Scarfone [65], organisations should, theoretically, immediately deliver security updates to minimise the time period in which the system remains vulnerable. Nevertheless, this is not possible due to the limited resources. As expressed by K. Christidis and M. Devetsikiotis [66], the current software updates distribution model (through a centralised server) for massively deployed IIoT devices may entail high maintenance costs. As pointed out by the authors, a peer-to-peer Blockchain based solution could be used to address this problem. For this purpose, a smart contract may be defined, which specifies how the software update will be deployed and applied [67]. In this line, *J. Li* proposed *Revere* [68,69], a self-organising, large-scale and resilient overlay network built on top of the Internet, which aims at rapidly disseminating security updates at high scale.

**Figure 10:**
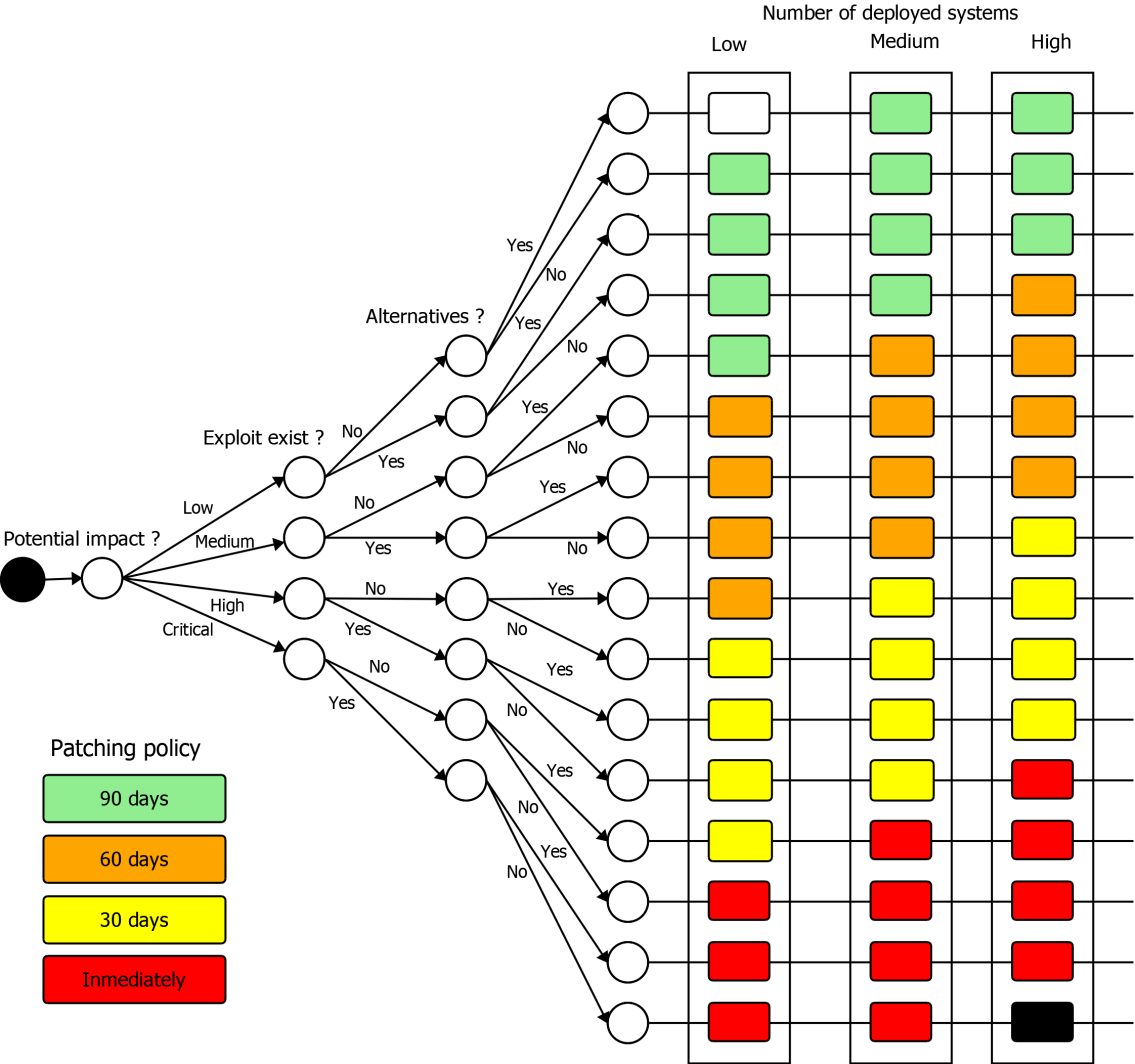
Software patch evaluation and delivery policy decision tree proposal.

Organisations commonly classify patches to be evaluated and delivered within a given time period, for example, 30 days, 60 days or 90 days. Figure 10 shows a software update delivery decision tree proposal, based on the software updates delivering factors specified by IEC 62443-4-1 [39].

The decision tree depicted in Figure 10 provides a guidance for a software update delivery policy. Firstly, the potential impact of the discovered vulnerability should be checked. To this end, the well-known Common Vulnerability Scoring System (CVSS) could be used (https://www.first.org/cvss/calculator/3.1). Based on the computed scoring, the potential impact is determined. This analysis has already been performed in the *Assessing* security issue management phase. The exploitation of such vulnerability and possible remediation alternatives (previously evaluated in the *Addressing* phase) should be examined afterwards. Lastly, the number of deployed systems should be considered.

It is worth mentioning that the study performed by B. Wang et al. [70] over 100 thousand industrial control devices showed that 50% of them where patched within the 60 days from the time of discovery of the vulnerability. As claimed by M. Souppaya and K. Scarfone [65]’s software patch testing, prioritisation and timings are frequently in conflict.

## 7. Conclusions

Security is becoming an essential feature to be addressed in the IIoT landscape. Industrial control systems should provide resiliency and protection against non-intentional and wilful cyber-attacks. As claimed by *Kaspersky* [1], “the number of vulnerabilities in ICS components keeps growing", which are widely diversified among vendors and products. Asset owners should keep track of new detected vulnerability and threats, and apply the corresponding measures to ensure the cyber-security capabilities of IIoT products throughout their entire lifespan.

In this article, the IEC 62443 industrial security standard [16] is analysed, focusing on cyber-security maintenance processes and procedures for IIoT systems and components. Three different stakeholders are defined, the asset owner, the service provider and the product supplier. Cyber-security shall then jointly and collaboratively be addressed by these entities, which are, first and foremost, highly advised to implement and enforce the ISO 27000 framework [17,18] and quality management [20,21] processes and procedures. Following IEC TR 63069 [13] and IEC TR 63074 [14], the defined security processes should be compatible with the safety processes, specifically, with the *overall operation, maintenance and repair* and *overall modification and retrofit* requirements established by IEC 61508 [11,15] (or with other safety-related maintenance processes described in any domain-specific standard, such as automotive [71]).

## 8. Future Lines and Open Issues

Although necessary, the security maintenance management of IIoT systems and components is a challenging task to be addressed in the current Industry 4.0 landscape. The required effort and resources for a successful and efficient IIoT systems and components maintenance program may vary significantly across domains (i.e., energy, transport) and markets. Therefore, as a precautionary measure, an adaptable and expandable maintenance program should be put in place. The use of security issues and software patches management and tracking tools are also highly recommended. In this line, further research and analysis on security-related events and incidents across all sectors is needed, in which time and resources required to address such issues is considered.

Beyond security issues and software updates management processes implemented by organisations, cyber-resilience features and capabilities might be considered for IACS. “Cyber-resilience refers to the ability of the system to prepare, absorb, recover and adapt to adverse effects, especially those associated with cyber-attacks” [72]. These properties may be of high relevance when an organisation is not able address and fix the encountered issue in a timely and efficient manner, for example, when a massive DDoS attack takes place. The IACS should be then able to absorb, recover and adapt to the new hostile circumstances. The MITRE corporation provides a general reference on cyber resilience metrics, measures and scoring scheme for systems and missions [73]. These metrics are usually based on a temporal model of disruptions and recovery of the system.

Finally, the timely distribution of software updates for massively deployed IIoT systems might be challenging. Usually, a central software updates repository is employed. In some other cases, the security update window might also be limited (i.e., connection to the server). Therefore, following the observations gleaned by K. Christidis and M. Devetsikiotis [66], H. Dai et al. [67] and J. Li [68,69], a robust, efficient and secure peer-to-peer software updates distribution scheme is needed.

## Figures and Tables

**Table 1:** Security levels (SL) defined by IEC 62443 [16].

Security Level	Target	Skills	Motivation	Means	Resources
SL 1	Casual or coincidental violations	No attack skills	Mistakes	Non-intentional	Individual
SL 2	Cybercrime	Generic	Low	Simple	Low (isolated individual)
SL 3	Hacktivist, terrorist	ICS specific	Moderate	Sophisticated (attack)	Moderate
SL 4	National state	ICS specific	High	Sophisticated (campaign)	Extended

**Table 2:** North American Reliability Corporation (NERC) Critical Infrastructure Protection (CIP) cyber security maintenance documents.

Code	Name
CIP-002-05	Categorisation of cyber systems of Bulk Electric System (BES)
CIP-003-05	Security Management Controls
CIP-004-05	Personnel and training
CIP-007-05	System Security Management
CIP-008-05	Incident Reporting and Response Planning
CIP-009-05	Recovery Plans for BES Cyber Systems
CIP-010-01	Configuration Change Management and Vulnerability Assessments

**Table 3:** Mapping of IEC 62443-2-X contents [35,36,37] to national standards (USA and Europe).

Country	Organisation	Standard	System Requirements for Security Management (IEC 62443-2-1)	Implementation Guidance for Information Security Management System (IEC 62443-2-1)	Patch Management (IEC 62443-2-3)	Requirements for Installation and Maintenance for Suppliers (IEC 62443-2-4)
USA	NIST	SP800-82	X	X	X	
	NERC	CIP-002-5	X			
		CIP-003-5	X	X		
		CIP-004-5		X		
		CIP-007-5		X		
		CIP-008-5		X		
		CIP-009-5		X		
		CIP-010-2	X	X		X
	Homeland security	CRR		X		
Germany	BSI	BSI Standard 100-1	X	X	X	
		BSI Standard 100-2	X	X	X	
		BSI Standard 100-3		X		
		BSI Standard 100-4		X		
Norway	OLF	OLF 104:2009		X		X
France	ANSSI	ANSSI Guides	X			
Spain	CCN-CERT	ENS	X	X	X	X
The Netherlands	TNO	TNO Guides	X			

**Table 4:** Mapping ISO 30111 [44] and IEC 62443-4-1: Practice 6 [16].

ISO 30111	IEC 62443-4-1: Practice 6
Preparation	
Receipt	Receiving (DM-1)
Verification	Reviewing (DM-2)
	Assessing (DM-3)
Remediation development	Addressing (DM-4)
Release	Disclosing (DM-5)
Post-release	

**Table 5:** Patch lifecycle states defined by IEC 62443-2-3 [36].

Patch State	Patch State Definition	Conducted by
Available	The patch has been provided by a third party or an IACS supplier but has not been tested	Asset owner/Product supplier
In test	The patch is being tested by the product supplier development team	Product supplier
Not approved	The patch has failed the testing and should not be used, unless and until the patch has been *Approved*	
Not applicable	The patch has been tested and is not considered relevant to IACS use	
Approved	The patch has passed testing	
Released	The patch is released for use by the product supplier or third party, or the patch may be directly applicable by the asset owner	Asset owner/Product supplier
In internal test	The patch is being tested by the asset owner	Asset owner
Not authorised	The patch has failed internal testing, or may not be applicable	
Authorised	The patch is released and meets company standards for updatable devices, or by inspection did not need testing	
Effective	The patch is posted for use	
Installed	The patch is installed on the system	
